# Epiafzelechin, a Flavanol, Regulates Lipid Homeostasis Through Modulation of HMGCR, PCSK9, and PPAR‐*α*: Mechanistic Insights and Therapeutic Implications

**DOI:** 10.1155/cdr/9082023

**Published:** 2026-01-28

**Authors:** Saud O. Alshammari, Nazifa Shahzad, Muhammad Nasir Hayat Malik, Qamar A. Alshammari, Abdulkarim Alshammari, Bassam S. M. Al Kazman, Muhammad Atif, Gideon F. B. Solre

**Affiliations:** ^1^ Department of Pharmacognosy and Alternative Medicine, College of Pharmacy, Northern Border University, Rafha, Saudi Arabia, nbu.edu.sa; ^2^ Faculty of Pharmacy, The University of Lahore, Lahore, Pakistan, uol.edu.pk; ^3^ Department of Pharmacology and Toxicology, College of Pharmacy, Northern Border University, Rafha, Saudi Arabia, nbu.edu.sa; ^4^ Department of Pharmacy Practice, College of Pharmacy, Northern Border University, Rafha, Saudi Arabia, nbu.edu.sa; ^5^ Department of Pharmacognosy, College of Pharmacy, Najran University, Najran, Saudi Arabia, nu.edu.sa; ^6^ Department of Chemistry, Thomas J. R. Faulkner College of Science and Technology, University of Liberia, Monrovia, Montserrado County, Liberia, ul.edu.lr

**Keywords:** docking, Epiafzelechin, HMGCR, hyperlipidemia, network pharmacology, PCSK9, PPAR-*α*, rats

## Abstract

Hyperlipidemia remains a leading modifiable risk factor for cardiovascular morbidity and mortality. Statins are considered the cornerstone of treatment; however, their adverse effects and limited efficacy in certain patient populations necessitate exploration of novel therapeutic avenues. Epiafzelechin (EZN), a flavanol with established antioxidant and anti‐inflammatory properties, was investigated for its potential role in lipid metabolism using an integrative approach combining network pharmacology, molecular docking, and in vivo validation. Putative EZN targets were predicted through SuperPred, Way2Drug, and PharmMapper, and intersected with hyperlipidemia‐related genes from GeneCards, DisGeNET, and CTD. Overlapping genes were subjected to protein–protein interaction (PPI) mapping, hub gene identification, and pathway enrichment analysis. Molecular docking was conducted to assess the binding affinity of EZN to lipid‐regulating proteins. Therapeutic efficacy of EZN was also evaluated in a TWR‐1339‐induced hyperlipidemic rat model using biochemical assays and real‐time PCR for gene expression profiling. A total of 105 genes were identified, involved in lipid transport, inflammatory signaling, and metabolic regulation. Functional enrichment and PPI analysis highlighted HMGCR, PCSK9, PPAR‐*α*, and LDLR as key targets. Docking studies revealed that EZN has strong binding affinities with these targets, supporting the structural feasibility of these interactions. In vivo, EZN treatment significantly reduced total cholesterol, triglycerides, LDL, and VLDL levels, while increasing HDL. Compared with simvastatin, EZN exhibited superior lipid‐lowering effects with a more favorable liver enzyme profile. Gene expression and ELISA analyses indicated downregulation of HMGCR, PCSK9, and APOB, and upregulation of PPAR‐*α*, LDLR, and SRB, highlighting its multi‐target modulation of lipid homeostasis. These findings indicate that EZN exerts broad regulatory effects on lipid metabolism through pleiotropic mechanisms and may represent a promising natural candidate for managing hyperlipidemia.

## 1. Introduction

Hyperlipidemia, characterized by elevated levels of lipids in the bloodstream, is regarded as a public health concern due to its association with cardiovascular diseases like atherosclerosis, heart attacks, and strokes [1–3]. The World Health Organization reports that billions of people around the globe suffer from dyslipidemia, with a large proportion of individuals residing in developed countries [4]. The major factors that contribute to the rise in hyperlipidemia include sedentary lifestyles, poor dietary habits, obesity, and genetic predispositions [5–7]. This high prevalence rate requires effective management strategies to mitigate cardiovascular risks and improve overall health outcomes [8].

Current treatment options for hyperlipidemia primarily include pharmacological agents such as statins, fibrates, and lifestyle modifications [9, 10]. Statins, including “Simvastatin and Atorvastatin”, are the most commonly prescribed medications because of their proven efficacy in lowering cholesterol levels and reducing the incidence of cardiovascular events [11, 12]. However, despite their widespread use, statins are associated with several adverse effects such as myopathy, liver enzyme elevation, and gastrointestinal disturbances, leading to poor patient compliance. Additionally, a significant number of patients fail to achieve target lipid levels, reiterating the need for alternative safer and efficacious therapies [13–15].

In recent years, there has been growing interest in natural compounds as potential therapeutic agents for hyperlipidemia. One such compound, Epiafzelechin (EZN), is a flavanol found in various plants including cocoa and green tea [16]. Flavanols, a common class of secondary plant metabolites, offer numerous health benefits by functioning as antioxidants, anticarcinogens, cardioprotective agents, antimicrobial, antiviral, and neuroprotective agents [17]. Studies indicate that EZN possess several beneficial properties, including bone‐protective, anti‐inflammatory, anticancer, and antioxidant effects, which make it a compound of interest for further exploration in the management of various disorders [18].

Given the limitations of current pharmacological treatments and the increasing interest in plant‐based bioactive compounds, our study aimed to explore the therapeutic potential of EZN in the management of hyperlipidemia. Utilizing an integrative approach, we employed network pharmacology to predict the molecular targets and pathways through which EZN may exert its lipid‐lowering effects. To validate these predictions, we conducted in vivo experiments to assess the anti‐hyperlipidemic efficacy of EZN in rat model. This comprehensive investigation provides novel insights into the mechanistic actions of EZN and supports its potential as a natural therapeutic agent for hyperlipidemia.

## 2. Materials and Methods

### 2.1. Network Pharmacology: EZN and Hyperlipidemia

#### 2.1.1. Data Collection

In this study, the identification of targets for EZN was conducted through several reputable databases (SuperPred, Way2Drug, and PharmMapper). SuperPred is known for its predictive capabilities regarding drug–target interactions, allowing for a comprehensive collection of potential targets. Way2Drug provides insights into drug mechanisms and associated biological targets, while PharmMapper focuses on identifying potential drug–target interactions based on pharmacophore mapping. Together, these databases enabled a robust compilation of targets relevant to EZN [19].

For the identification of genes associated with hyperlipidemia, we accessed multiple databases, including DisGeNET, GeneCards, and the Comparative Toxicogenomics Database (CTD). DisGeNET is a comprehensive platform that aggregates genetic associations with diseases, facilitating the extraction of relevant hyperlipidemia‐related genes. GeneCards offers a wealth of information on human genes, including their functions and associations with various conditions. The CTD provides insights into the effects of environmental chemicals on human health, further enriching our dataset with genes linked to hyperlipidemia [20–22].

#### 2.1.2. Data Processing

Following the collection of data, we performed ID mapping by employing UniProt database. This step was crucial for standardizing gene identifiers across the multiple data sources, ensuring consistency and accuracy in our analysis. UniProt is a well‐validated resource that provides comprehensive protein sequence and functional information. After mapping the identifiers, we removed duplicate entries to maintain a unique set of gene targets for both EZN and hyperlipidemia. This process was essential to avoid redundancy and enhance the reliability of subsequent analyses [23].

#### 2.1.3. Common Gene Identification

To identify the intersection of gene targets between EZN and hyperlipidemia, we constructed a Venn diagram using the SR plot. This visual representation allowed for a clear understanding of the common genes shared between the two datasets. The Venn diagram facilitated the identification of overlapping targets, highlighting genes that may play a significant role in the pharmacological effects of EZN on hyperlipidemia [24].

#### 2.1.4. Protein–Protein Interaction (PPI) Network

The common genes identified from the Venn diagram were introduced into the STRING database to generate a PPI network. STRING is a well‐established tool that provides insights into the interactions between proteins based on known and predicted associations. The resulting PPI network offers a comprehensive view of how the common genes interact at a molecular level, which is critical for understanding the biological mechanisms underlying the pharmacological effects of EZN. To visualize the PPI network effectively, we imported the data into Cytoscape, a powerful software platform for network analysis and visualization. Cytoscape enables the exploration of complex networks and facilitates the identification of key nodes and interactions within the network [25, 26].

#### 2.1.5. Hub Gene Identification

Hub genes are often critical for understanding disease mechanisms and potential therapeutic targets. For further analysis of PPI network, we utilized the Degree method within the CytoHubba plugin in Cytoscape. This method identifies hub genes that play a central role in the network based on their connectivity [27, 28].

#### 2.1.6. Functional Enrichment Analysis

Finally, we conducted functional enrichment analyses to gain insights into the biological implications of the identified genes. For this purpose, we employed ShinyGO, a user‐friendly tool for Gene Ontology (GO) analysis. This analysis covered three main categories: Biological Process (BP), Cellular Component (CC), and Molecular Function (MF). By categorizing the genes, we aimed to elucidate their roles in various biological contexts. Additionally, we performed KEGG pathway analysis to explore the pathways associated with the common genes. KEGG pathways provide valuable information on the biochemical pathways and processes in which the identified genes are involved, allowing us to understand the broader biological implications of EZN’s effects on hyperlipidemia [29].

### 2.2. Molecular Docking

#### 2.2.1. Protein Structure Preparation

The 3D structures of target proteins were sourced from the Protein Data Bank (PDB) via the Research Collaboratory for Structural Bioinformatics (RCSB). The MOE Protonate‐3D module facilitated the preprocessing of these protein structures for docking studies. This process involved removing co‐crystallized ligands, water molecules, and other heteroatoms, as well as adding missing hydrogen atoms and optimizing side‐chain orientations. To minimize energy and address any steric clashes or structural flaws, the protein structures underwent further refinement using the AMBER99 force field [30].

#### 2.2.2. Preparation of Ligand

The ligand structure was retrieved from the PubChem database maintained by NCBI. Using BIOVIA Discovery Studio, this structure was converted to PDB format for additional analysis [31]. This conversion allowed for the visualization and exploration of the ligand’s 3D structure from public databases. Subsequently, MOE preprocessed the ligand molecule by eliminating counterions and salts to establish the correct protonation state. Finally, the ligand’s energy was minimized using the MMFF94x force field to achieve its most stable conformation [32].

#### 2.2.3. Molecular Docking

Molecular docking and scoring were conducted using MOE version 2019.0102. The docking study involved ligands interacting with various protein targets and was visualized with the BIOVIA Discovery Studio Visualizer. Initially, a ligand database was created in MOE and converted into a Microsoft Access database file (MDB format). The input files were then uploaded for docking analysis, utilizing MOE’s default docking algorithm with 100 ligand conformations. The docking employed the Triangle Matcher algorithm, with London dG and GBVI/WSAdG serving as scoring functions. The optimal docking result was chosen based on conformational visualization in BIOVIA [33].

### 2.3. In Vivo Study

#### 2.3.1. Animals

Male Sprague–Dawley rats (6–8 weeks old, weighing between 170 and 200 g) were acquired from the University of Veterinary and Animal Sciences, Lahore, Pakistan, and were housed in the animal care facility of Faculty of Pharmacy, The University of Lahore under standard conditions. To allow them to acclimate, the rats were given a week to adapt to their new environment. The care provided to the animals adhered to OECD norms [34]. Additionally, the experimental protocol was approved by the Institutional Research Ethics Committee, Faculty of Pharmacy, University of Lahore (Approval number: IREC‐2024‐17) [35].

#### 2.3.2. Hyperlipidemia Induction

Hyperlipidemia was induced by a single intraperitoneal dose (400 mg/kg) of TWR‐1339 (Sigma Aldrich, United States) in rats and 24 h post‐induction, animals in disease group were daily treated with triton X‐100 while treated groups received daily dose (200 mg/kg) of triton X‐100 (Sigma Aldrich, United States) with simvastatin (SIM; 40 mg/kg) or various doses of EZN (50 and 100 mg/kg) for 7 days. Upon completion of the study period, animals were euthanized with pentobarbital sodium (200 mg/kg) and killing was ensured by cervical dislocation. Blood samples were collected via cardiac puncture in gel tubes and subsequently analyzed for lipid profile and liver function test (LFT) analyses. Liver samples were collected for real‐time polymerase chain reaction (PCR) and ELISA assays [36].

#### 2.3.3. Study Design

Animals were divided into five groups with three animals in each group:

1. Normal control: Received no treatment
2. TWR‐1339 (400 mg/kg): Received single dose of TWR‐1339 (400 mg/kg) followed by triton X‐100 (200 mg/kg) for 7 days
3. SIM (20 mg/kg): Received daily doses of SIM and triton X‐100 for 7 days
4. EZN (50 mg/kg): Received daily doses of EZN (50 mg/kg) and triton X‐100 for 7 days
5. EZN (100 mg/kg): Received daily doses of EZN (100 mg/kg) and triton X‐100 for 7 days

#### 2.3.4. Lipid Profile and LFT Analysis

At the conclusion of the experiment, the animals were humanely euthanized. Prior to decapitation, the rats were sedated, and blood samples were collected into sterile, dry tubes. The samples were then centrifuged for 10 min to separate the serum, which was subsequently used for biochemical analysis. Serum levels of TC, TG, LDL, HDL, AST, and ALT were measured using a standard commercial assay kit (BioLabs, Boston, United States) [37].

### 2.4. RNA Extraction, cDNA Synthesis, and Quantitative Real‐Time PCR

Total RNA was isolated from tissue samples using TRIzol reagent (Invitrogen) following the manufacturer’s protocol. Briefly, samples were homogenized in TRIzol, and phase separation was achieved by adding chloroform, followed by centrifugation. The aqueous phase containing RNA was carefully transferred to a fresh tube and precipitated with isopropanol. After washing with 75% ethanol, the RNA pellet was air‐dried and resuspended in RNase‐free water. The concentration and purity of the extracted RNA were assessed using a NanoDrop spectrophotometer (Thermo Fisher Scientific, United States).

Subsequently, 1 *μ*g of total RNA was reverse‐transcribed into complementary DNA (cDNA) using the WizScript cDNA Synthesis Kit (WizBio Solutions, New Mexico, United States), according to the manufacturer’s instructions. Quantitative real‐time PCR was performed using the StepOne Real‐Time PCR System (Applied Biosystems) in combination with the Zokeyo 2× SYBR Green qPCR Mixture (Zokeyo, China). Each reaction was carried out in a total volume of 20 *μ*L containing cDNA template, gene‐specific primers, and SYBR Green master mix. Expression levels of target genes were quantified using the comparative Ct (*ΔΔ*Ct) method. Hypoxanthine–guanine phosphoribosyltransferase (HPRT) was used as the internal reference gene to normalize the expression data, ensuring accurate and reliable quantification of relative transcript levels.

### 2.5. Statistical Analysis

Data from three biological replicates were presented as mean ± standard deviation. Statistical analysis was performed by one‐way ANOVA, followed by Tukey’s multiple comparison test, using GraphPad Prism version 8.02. A *p* value of less than 0.05 was considered statistically significant. The levels of significance were indicated as follows: ∗∗∗*p* ≤ 0.001, ∗∗*p* ≤ 0.01, and ∗*p* ≤ 0.05.

## 3. Results

### 3.1. Target Identification and Gene Mapping

**Figure 2:**
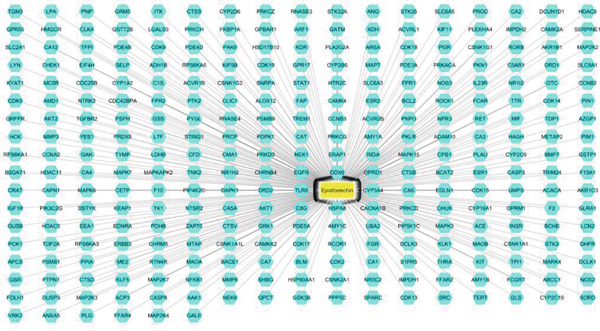
Compound‐target network of EZN. The central yellow node represents EZN, while the surrounding cyan nodes indicate the associated genes.

The initial phase of our analysis involved the identification of potential targets for EZN using three distinct databases: Way2Drug (65), SuperPred (116), and PharmMapper (129). As illustrated in Figure 1a, a total of 310 potential targets were identified from these sources. Following the integration of these targets, we employed the UniProt database for ID mapping, which allowed us to standardize gene identifiers. After this step, we processed the data to remove duplicates, resulting in a refined list of 295 unique targets for EZN (Figure 2). Simultaneously, genes associated with hyperlipidemia were extracted from DisGeNET (8), GeneCards (1384), and the CTD (170). This effort yielded 1995 unique genes related to hyperlipidemia. The integration of these datasets allowed us to identify common genes shared between the targets of EZN and those implicated in hyperlipidemia. The analysis revealed that 105 genes were common to both the EZN targets and hyperlipidemia‐related genes. This significant overlap suggests potential therapeutic pathways through which EZN may influence hyperlipidemia (Figures 1, 2).

Figure 1Overview of the target identification process for EZN and the common genes associated with hyperlipidemia. (a) Flow chart illustrates the hub genes selection via various data bases. (b) The Venn diagram illustrates the overlap between EZN targets (190) and hyperlipidemia‐related genes (1886), highlighting 105 common genes.

**Figure:**
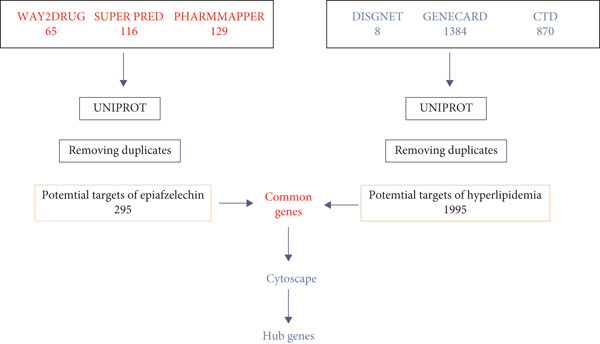
(a)

**Figure:**
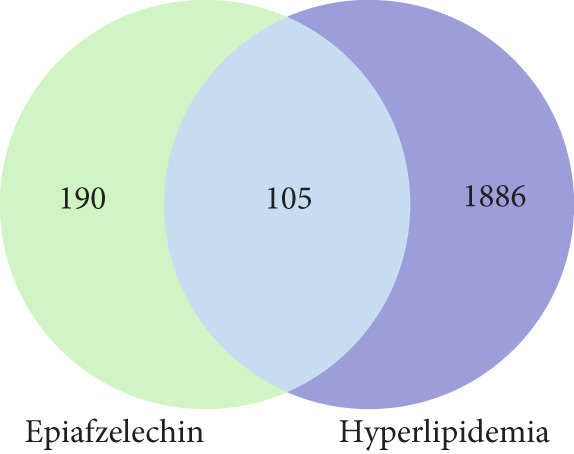
(b)

### 3.2. PPI Network

The PPI network illustrates the complex interactions among the 105 common genes identified in the study. This network comprises 104 nodes (representing genes) and 798 edges (representing interactions), indicating a robust set of relationships among the proteins encoded by these genes. The average node degree of 15.3 signifies that, on average, each gene interacts with approximately 15 other genes within the network. Additionally, the average local clustering coefficient of 0.522 suggests that there is a moderate tendency for the proteins to form clusters, implying that some genes may work together in specific biological pathways or functions.

The PPI enrichment *p* value of < 1.0e−16 indicates that the observed interactions are significantly more numerous than would be expected by chance. This strong statistical significance suggests that these genes are likely to be involved in coordinated biological processes, reinforcing the hypothesis that EZN may exert its effects on hyperlipidemia through these interconnected pathways (Figure 3a).

Figure 3PPI network analysis. (a) Protein–protein interaction (PPI) network of common genes associated with EZN, illustrating the complex interactions among 105 shared genes. (b) Hub gene analysis highlighting key nodes (in red and orange) that exhibit high connectivity within the network, suggesting their potential importance in mediating the effects of EZN on hyperlipidemia.

**Figure:**
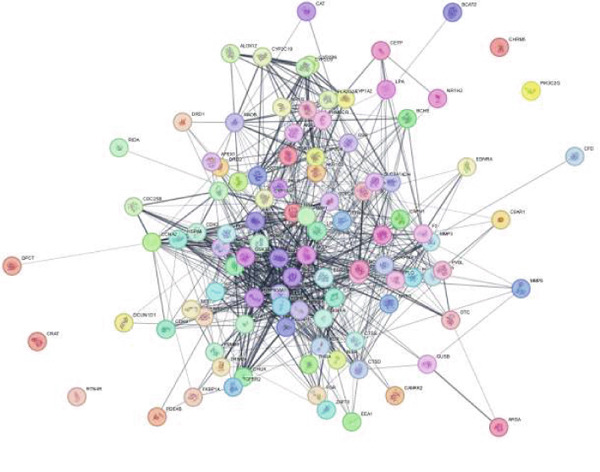
(a)

**Figure:**
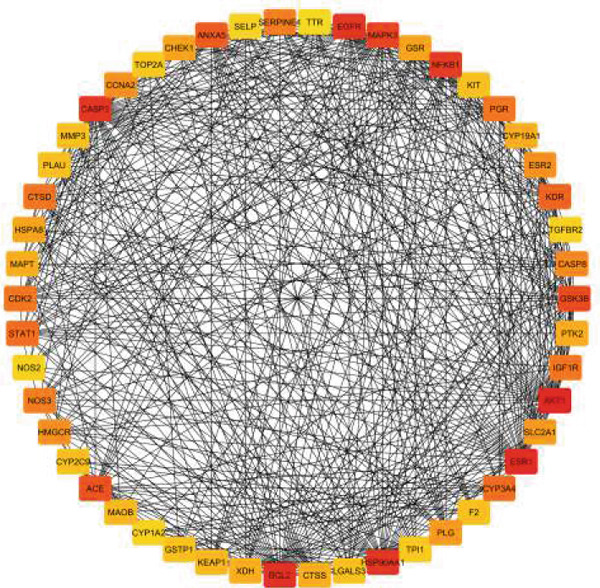
(b)

### 3.3. Hub Genes Analysis

In the adjacent image, the top 50 hub genes within the PPI network are highlighted. Hub genes are defined as those with a high degree of connectivity, meaning they interact with a larger number of other genes compared with non‐hub genes. The identification of these hub genes is crucial, as they often play key roles in regulating biological processes and can serve as potential therapeutic targets (Figure 3b).

### 3.4. Functional Enrichment Analysis

The functional enrichment analysis conducted on the common genes identified in this study provides valuable insights into their roles in relation to hyperlipidemia and the pharmacological effects of EZN. This analysis is categorized into three main GO domains: BP, CC, and MF, alongside a KEGG pathway analysis, each shedding light on different aspects of gene functionality and interaction. In the BP category, the analysis revealed that several key processes were significantly associated with the common genes. A major finding was the involvement of these genes in responses to various stimuli, including both chemical and physical signals. This suggests that the proteins encoded by these genes play critical roles in how cells adapt to environmental changes, which is particularly relevant in metabolic conditions like hyperlipidemia. Moreover, the enrichment of genes associated with cellular metabolic processes underscores their importance in lipid metabolism and homeostasis. This indicates that they may participate in pathways that regulate lipid synthesis, degradation, and transport, highlighting a potential mechanism through which EZN may exert its beneficial effects on lipid profiles. The CC analysis provided insights into the spatial distribution of the identified genes. The results indicated that many common genes are located in extracellular regions, suggesting that their proteins may be involved in signaling pathways that influence lipid metabolism from outside the cell. Additionally, a significant number of genes were associated with cell membranes, which is critical for processes such as lipid uptake and transport. This spatial context reinforces the notion that these genes not only function within cells but also engage in essential interactions with the extracellular environment, potentially influencing cellular responses to lipid levels and metabolic cues. The MF analysis highlighted the specific biochemical activities of the proteins encoded by the common genes. Notably, many genes were found to exhibit receptor activity, indicating their roles in mediating cellular responses to hormones and other signaling molecules that regulate lipid metabolism. This suggests that these proteins may be integral to pathways that respond to changes in lipid levels or hormonal signaling, which is crucial for maintaining lipid homeostasis. Furthermore, the presence of genes with catalytic activities points to their direct involvement in metabolic reactions, reinforcing the idea that they contribute to the biochemical processes necessary for lipid processing and regulation.

**Figure 4:**
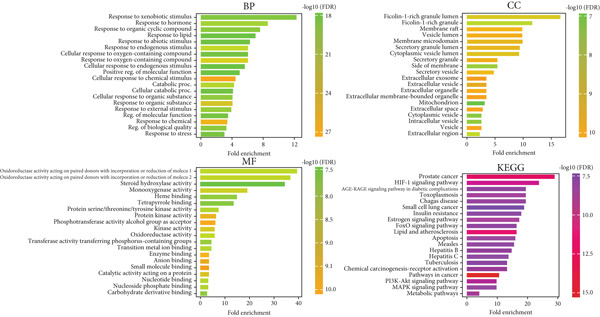
Functional enrichment analysis of common genes related to EZN. BP, CC, MF, and KEGG pathway analyses highlight significant processes and pathways associated with lipid metabolism and other relevant biological functions. Bar heights indicate field enrichment, with color gradients representing statistical significance (log10 *p* value).

The KEGG pathway analysis expanded the understanding of the biological implications of the identified genes within broader metabolic networks. Significant pathways were highlighted, including the AGE‐RAGE signaling pathway, which is known to be involved in the regulation of oxidative stress and inflammation—two critical factors in the context of hyperlipidemia. The identification of this pathway suggests that the common genes may not only influence lipid metabolism directly but also play roles in mediating inflammatory responses that can exacerbate metabolic disorders. Additionally, the analysis revealed connections to pathways associated with conditions such as prostate cancer, indicating that the effects of hyperlipidemia may extend beyond lipid metabolism to influence other chronic diseases, thereby providing a broader context for the therapeutic potential of EZN (Figure 4).

### 3.5. Docking Affinity of EZN and SIM With Target Proteins

**Figure 5:**
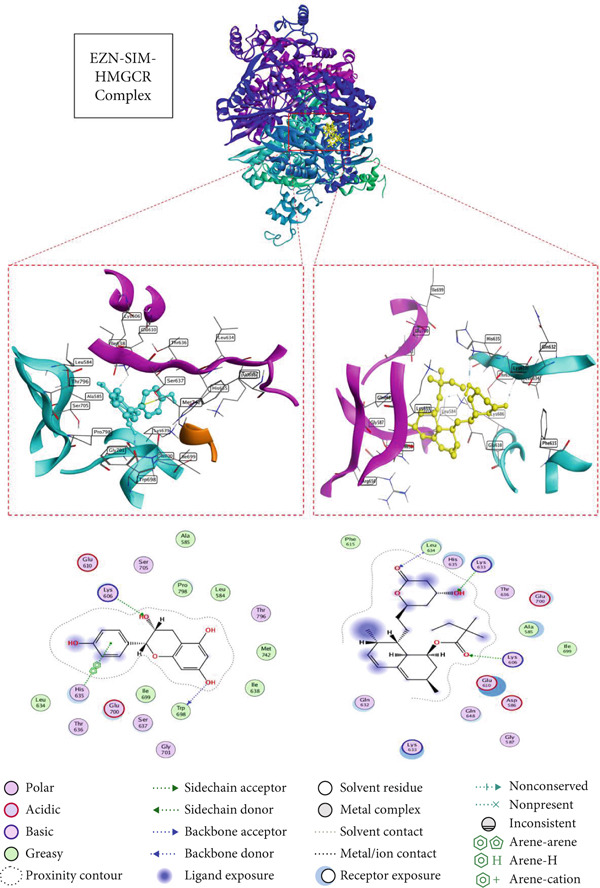
2D and 3D interactions of EZN (cyan) and SIM (yellow) with HMGCR.

**Figure 6:**
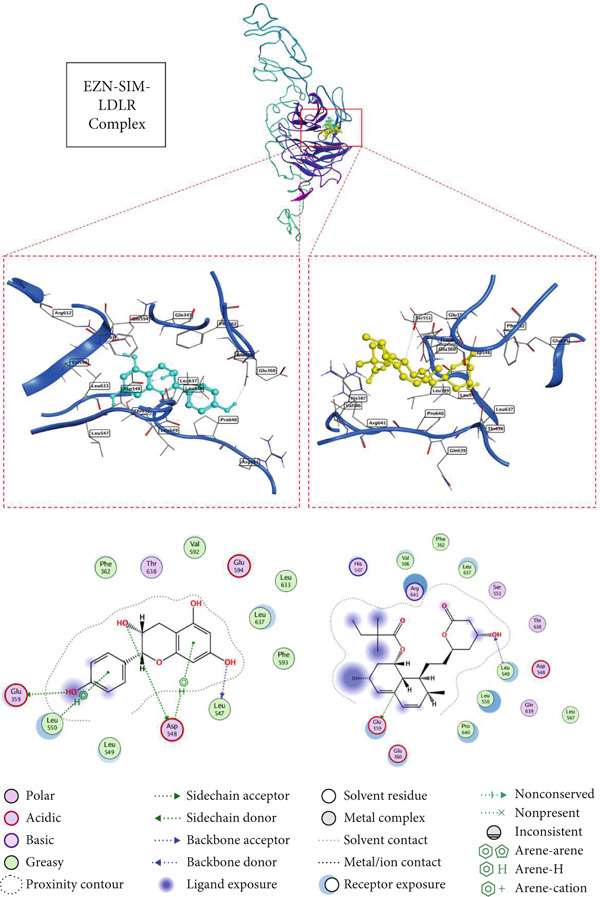
2D and 3D interactions of EZN (cyan) and SIM (yellow) with LDLR.

**Figure 7:**
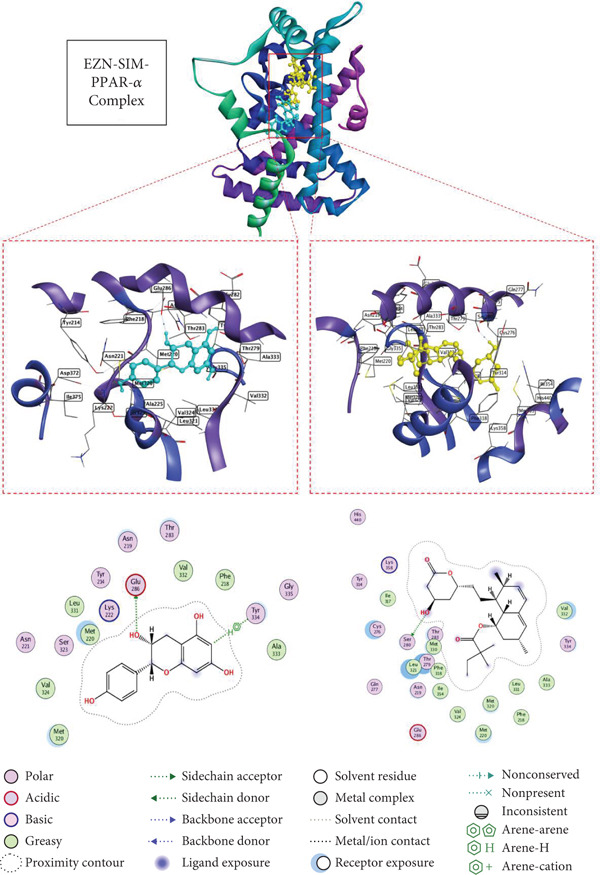
2D and 3D interactions of EZN (cyan) and SIM (yellow) with PPAR‐*α*.

**Figure 8:**
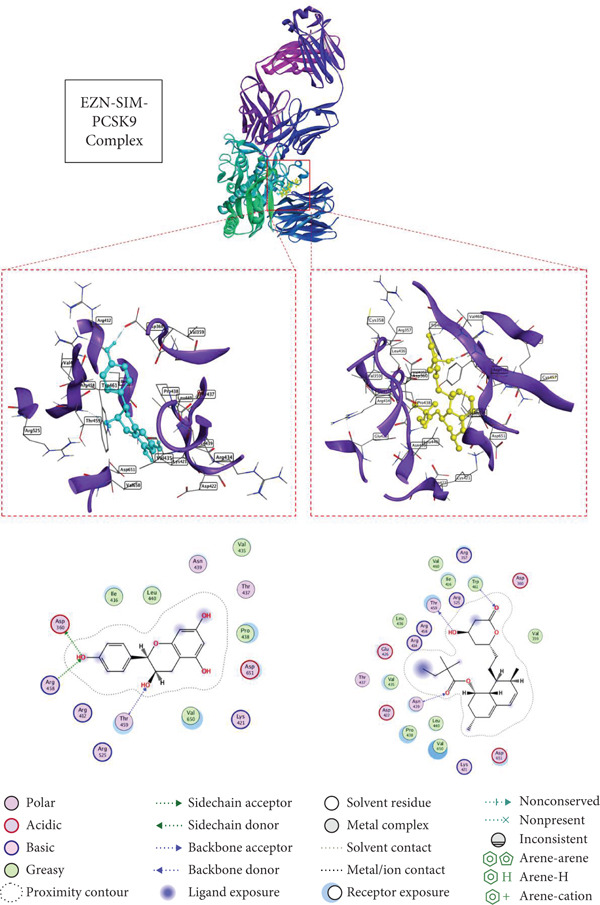
2D and 3D interactions of EZN (cyan) and SIM (yellow) with PCSK9.

The docking results for EZN, compared with the standard drug SIM, highlight its potential as an effective therapeutic agent for hyperlipidemia. In the case of HMGCR (PDB ID: 1HW8), EZN exhibited a binding score of −5.88 kcal/mol with an RMSD of 1.98 Å, forming significant hydrogen bonds with key residues TRP 698 and LYS 606 at distances of 2.77 and 3.12 Å, respectively. These interactions are critical for stabilizing the enzyme–inhibitor complex and suggest that EZN can effectively inhibit cholesterol biosynthesis, similar to SIM, which demonstrated a stronger binding score of −6.48 kcal/mol and lower RMSD. In the context of LDLR (PDB ID: 1N7D), EZN showed a binding score of −6.52 kcal/mol, interacting with multiple residues, including LEU 547 and GLU 359, through hydrogen bonds at distances of 2.83 and 3.48 Å. This affinity indicates a robust potential for modulating LDLR activity, essential for lipid clearance. SIM again outperformed with a score of −7.05 kcal/mol, but the close binding affinity of EZN suggests it may similarly enhance LDLR‐mediated lipid uptake. In PPAR‐*α* (PDB ID: 2ZNN), EZN scored −5.98 kcal/mol, forming hydrogen bonds with GLU 286 and TYR 334, which are crucial for transcriptional regulation of lipid metabolism. Lastly, in PCSK9 (PDB ID: 2H42), EZN achieved a binding score of −6.30 kcal/mol, engaging with ASP 360 and ARG 458 key residues that influence LDLR degradation (Table 1, Figures 5, 6, 7, and 8).

**Table 1:** Docking of EZN and SIM with target proteins.

**Compounds**	**S Score (kcal/mol)**	**RMSD (Å)**	**Atom of compounds**	**Atom of receptors**	**Residue of receptor**	**Type of interactions**	**Distance (Å)**	***E* value (kcal/mol)**
HMGCR (PDB ID: 1HW8)								
EZN	−5.8859	1.9883	O12 O20 6‐ring	O NZ 5‐ring	TRP 698 (C) LYS 606 (B) HIS 635 (B)	H‐donor H‐acceptor pi‐pi	2.77 3.12 3.96	−2.3 −0.6 −0.0
SIM	−6.4830	1.5362	O7 O27 O29	NZ N NZ	LYS 606 (C) LEU 634 (C) LYS 633 (C)	H‐acceptor H‐acceptor H‐acceptor	3.02 3.22 3.15	−0.7 −1.1 −3.1
LDLR (PDB ID: 1N7D)								
EZN	−6.5224	1.8285	O12 O19 O20 6‐ring 6‐ring 6‐ring	O OE1 OD1 CA CB CD2	LEU 547 (A) GLU 359 (A) ASP548 (A) ASP548 (A) LEU550 (A) LEU 550 (A)	H‐donor H‐donor H‐donor pi‐H pi‐H pi‐H	2.83 3.48 3.31 4.64 4.02 4.08	−1.8 −0.5 −0.9 −0.6 −0.5 −0.5
SIM	−7.0558	1.5853	C14 O29	OE1 N	GLU 359 (A) LEU 549 (A)	H‐donor H‐acceptor	3.18 2.99	−0.5 −1.8
PPAR‐*α* (PDB ID: 2ZNN)								
EZN	−5.9885	1.8165	O20 C8	OE1 6‐ring	GLU 286 (A) TYR 334 (A)	H‐donor H‐pi	3.28 4.05	−0.8 −0.5
SIM	−6.8678	1.4618	O29	OG	SER 280 (A)	H‐donor	2.81	−1.0
PCSK9 (PDB ID: 2H42)								
EZN	−6.3085	1.3480	O19 O19 O20	OD1 NH1 N	ASP 360 (B) ARG 458 (B) THR 459 (B)	H‐donor H‐acceptor H‐acceptor	2.84 3.19 3.22	−4.9 −0.5 −1.5
SIM	−7.0478	1.2622	O29 O7 O27	O N N	THR 459 (B) ASN 439 (B) TRP 461 (B)	H‐donor H‐acceptor H‐acceptor	3.06 2.78 2.87	−1.4 −2.3 −2.3

### 3.6. EZN Improved the Lipid Profile by Reducing TC, TG, LDL, VLDL Levels and Increasing HDL Levels

The administration of TWR‐1339 led to significant increase in serum lipid parameters including LDL, TG, TC, and VLDL, along with a notable reduction in HDL levels, indicating severe hyperlipidemia compared with the control group. Treatment with SIM (20 mg/kg) and both doses of EZN (50 and 100 mg/kg) significantly reversed these effects. LDL levels, which were markedly elevated in the TWR‐1339 group, were substantially reduced upon treatment, with both EZN doses showing strong efficacy. EZN at 50 mg/kg showed slightly lower LDL levels compared with the 100 mg/kg dose, indicating a slightly stronger effect. Similarly, TG levels were significantly decreased in the EZN‐treated groups compared with the TWR‐1339 group, with the 50 mg/kg dose of EZN showing a marginally better reduction than the 100 mg/kg dose. In the case of VLDL, both EZN doses significantly reduced the elevated levels induced by TWR‐1339. TC levels followed the same trend, where both EZN‐treated groups showed significant decrease compared with the TWR‐1339 group, with EZN 50 mg/kg showing marginally better results than 100 mg/kg. HDL levels, which were decreased due to TWR‐1339, were significantly increased by both doses of EZN and SIM. Overall, both 50 and 100 mg/kg doses of EZN effectively improved the lipid profile. However, EZN at 50 mg/kg demonstrated slightly better effects in reducing LDL, TG, VLDL, and TC levels, and increasing HDL levels (Figure 9).

Figure 9EZN improved lipid profile in TWR‐1339‐induced hyperlipidemic rats. TWR‐1339 induced significant dyslipidemia, characterized by elevated TC (a), TG (b), LDL (c), and VLDL (d) levels, and reduced HDL (e). Treatment with EZN effectively restored lipid parameters towards normal. At 50 mg/kg, EZN showed lipid‐lowering effects comparable with SIM, significantly improving the overall lipid profile. ∗∗∗*p* ≤ 0.001, ∗∗*p* ≤ 0.01.

**Figure:**
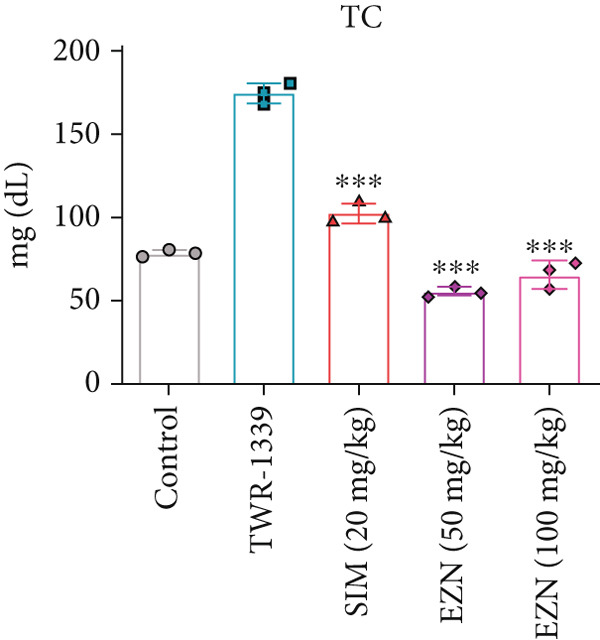
(a)

**Figure:**
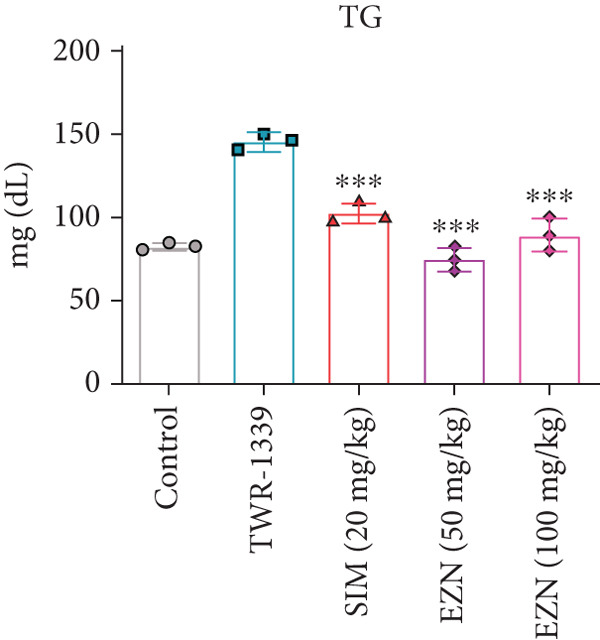
(b)

**Figure:**
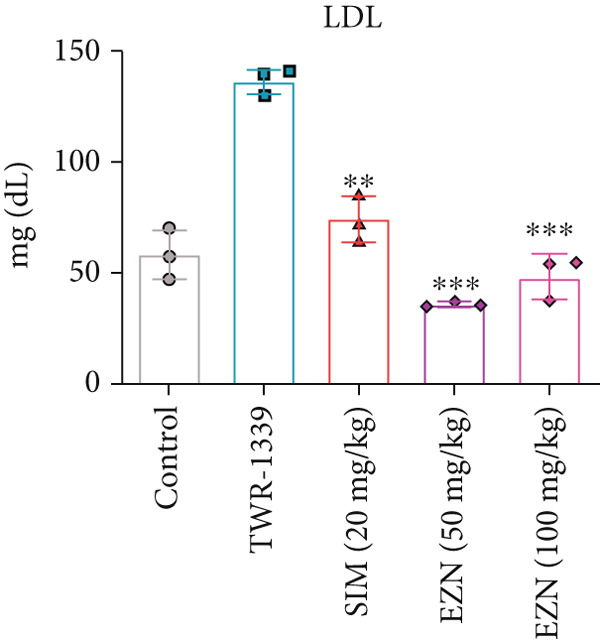
(c)

**Figure:**
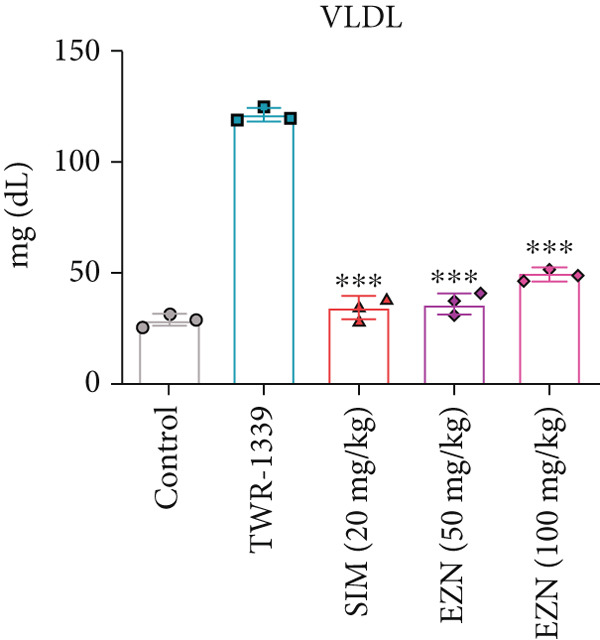
(d)

**Figure:**
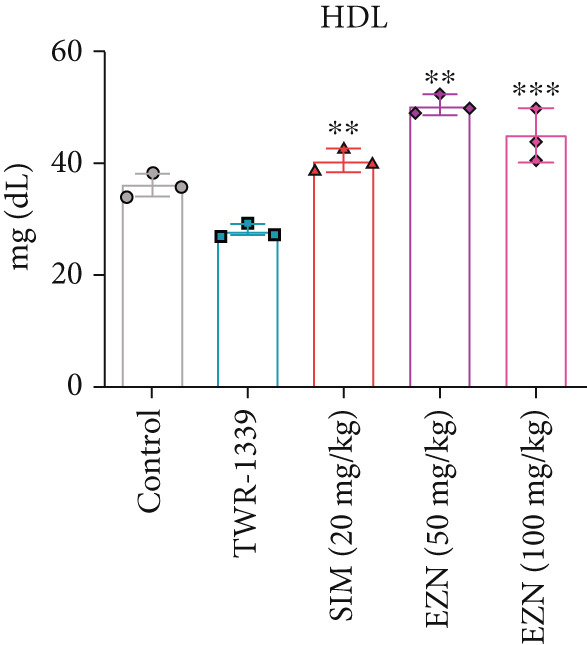
(e)

### 3.7. EZN Affected the Levels of Liver Function Enzymes (ALP, ALT, AST)

The AST levels in all treatment groups appeared to be higher than those in the control. TWR‐1339 and SIM‐treated groups displayed slight elevations, while EZN at 50 and 100 mg/kg significantly elevated AST levels, indicating a potential hepatic impact. However, no substantial difference was found between the two EZN doses, suggesting both exhibited similar effects in increasing AST levels. In the case of ALP, the SIM‐treated group showed a significant increase compared with the control and TWR‐1339 groups. EZN also caused a slight rise in ALP, with the 100 mg/kg dose resulting in a statistically significant increase. This suggests that EZN at 100 mg/kg dose had a more noticeable effect on ALP levels than the lower dose, which may indicate dose‐related hepatic stress at moderate levels. ALT levels in SIM group rose noticeably, although not statistically significant. EZN at both doses (50 and 100 mg/kg) showed reductions in ALT levels compared with SIM. Interestingly, EZN 50 mg/kg led to a slightly greater reduction in ALT levels than the 100 mg/kg dose. However, the changes in ALT remained statistically nonsignificant across groups. Overall, these findings suggest that EZN at 100 mg/kg may exert more prominent hepatotoxic effects in terms of ALP, while both doses similarly influence AST. The ALT‐lowering trend of EZN may indicate potential hepatoprotective properties, especially at the 50 mg/kg dose, but further investigation is needed to confirm statistical relevance (Figure 10).

Figure 10EZN did not induce ALT and ALP levels in hyperlipidemic rats. SIM demonstrated an induction in AST (a), ALT (b) and ALP (c) levels, while EZN treatment, particularly at 50 mg/kg, markedly reduced ALT and ALP levels toward normal, suggesting a hepatoprotective effect. ∗∗∗*p* ≤ 0.001, ∗∗*p* ≤ 0.01, ∗*p* ≤ 0.05.

**Figure:**
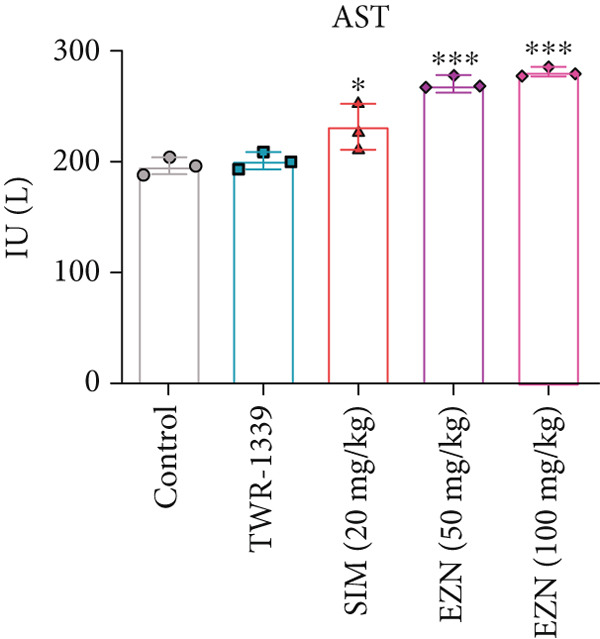
(a)

**Figure:**
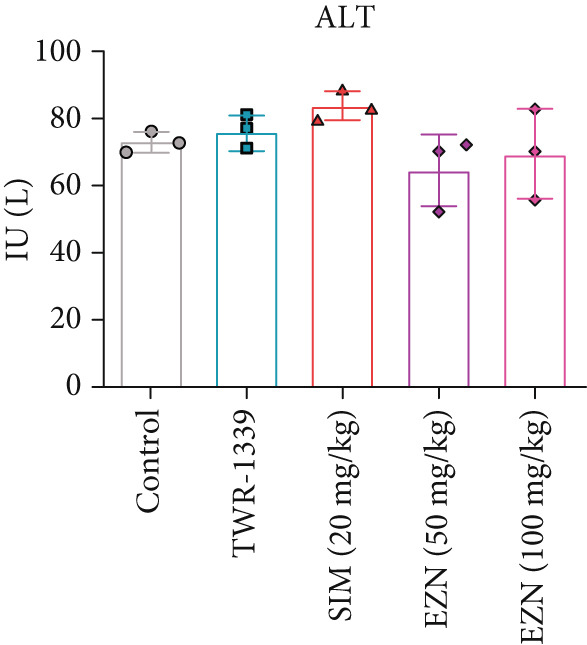
(b)

**Figure:**
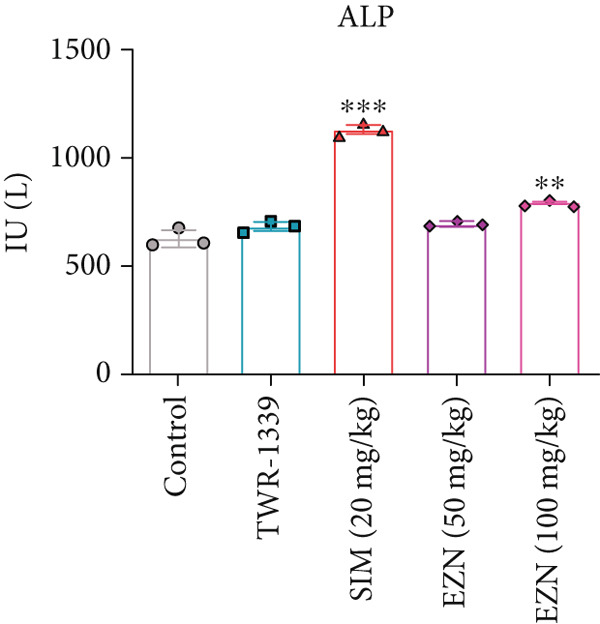
(c)

### 3.8. EZN Modulates the Expression of HMGCR, APOB, LDLR, PPAR‐*α*, PCSK9, and SRB

All EZN treatment groups exhibited significant modulatory effects on the expression of lipid metabolism‐associated genes, suggesting a strong influence of EZN on key regulatory pathways. The expression of PPAR‐*α* was markedly upregulated in both EZN 50 and 100 mg/kg groups, indicating a stimulatory effect. Though both doses significantly elevated PPAR‐*α* levels, a more prominent increase was observed at the higher dose, reflecting a dose‐dependent trend. The upregulation was statistically significant at both concentrations (*p* < 0.001 for 100 mg/kg; *p* < 0.01 for 50 mg/kg), suggesting EZN’s potent role in promoting lipid oxidation via PPAR‐*α* activation.

In contrast, PCSK9 expression was significantly downregulated in all EZN‐treated groups compared with the TWR‐1339‐induced model. EZN at both 50 and 100 mg/kg doses demonstrated robust inhibitory effects, with comparable levels of suppression. The reductions were highly significant (*p* < 0.001), and the minimal difference between doses indicates that the lower dose was sufficient to exert maximal PCSK9 downregulation.

The regulation of SRB (SR‐BI) was more nuanced. EZN 50 mg/kg significantly increased SRB expression (*p* < 0.01), suggesting an optimal stimulatory response at this dose. Although EZN 100 mg/kg also elevated SRB levels, the increase was not statistically significant, indicating a potential plateau or reduced efficacy at the higher dose.

**Figure 12:**
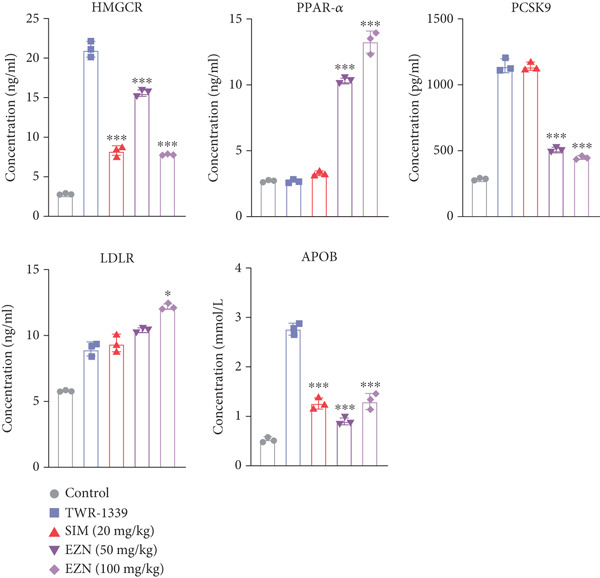
EZN differentially regulate lipid modulating proteins in TWR‐1339‐induced hyperlipidemia model. EZN downregulated HMGCR, PCSK9, and APOB while upregulated PPAR‐*α* and LDLR levels. ∗∗∗*p* ≤ 0.001, ∗*p* ≤ 0.05.

The expression of HMGCR was moderately elevated following EZN treatment, with both doses inducing upregulation compared with control. The increase was more pronounced at 100 mg/kg, though statistical significance was only achieved at the higher dose (*p* < 0.05), indicating a possible dose‐responsive effect on cholesterol biosynthesis. For LDLR, an increase trend in gene expression was observed in both EZN‐treated groups. This upregulation supports the beneficial role of EZN in promoting LDL clearance and maintaining lipid homeostasis. In contrast, APOB expression was significantly raised in TWR‐1339‐induced hyperlipidemic rats. Treatment with SIM and EZN significantly reduced the levels of APOB. Similar findings were witnessed in ELISA assay, in which a reduction in HMGCR, PCSK9, and APOB levels were observed while an upsurge in the levels of PPAR‐*α* and LDLR was observed (Figures 11 and 12).

Figure 11EZN treatment significantly modulated the expression of key lipid‐regulating genes in TWR‐1339‐induced hyperlipidemic rats. EZN downregulated PCSK9 (b), APOB (c) and HMGCR (d), indicating reduced cholesterol synthesis and lipid transport. Meanwhile, PPAR‐*α* (a), SRB (e), and LDLR (f) were upregulated, reflecting enhanced lipid oxidation and clearance. ∗∗∗*p* ≤ 0.001, ∗∗*p* ≤ 0.01.

**Figure:**
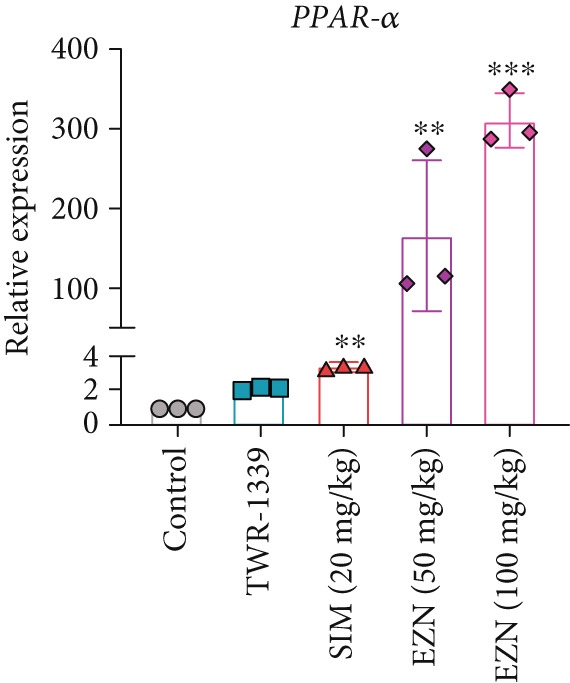
(a)

**Figure:**
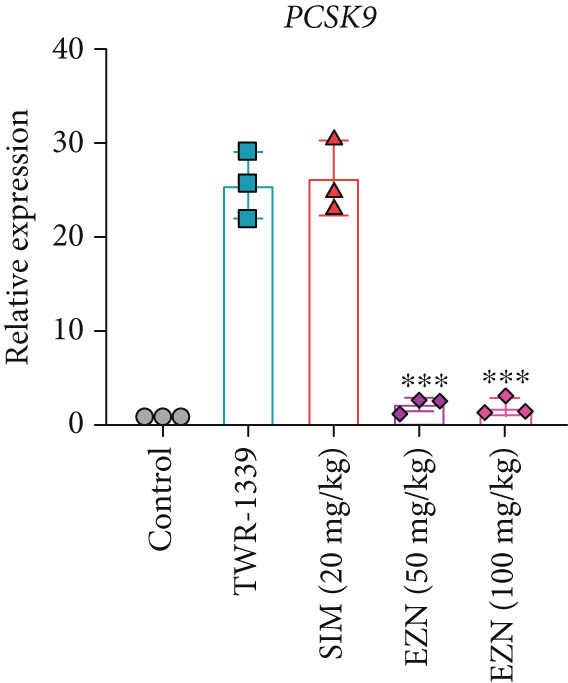
(b)

**Figure:**
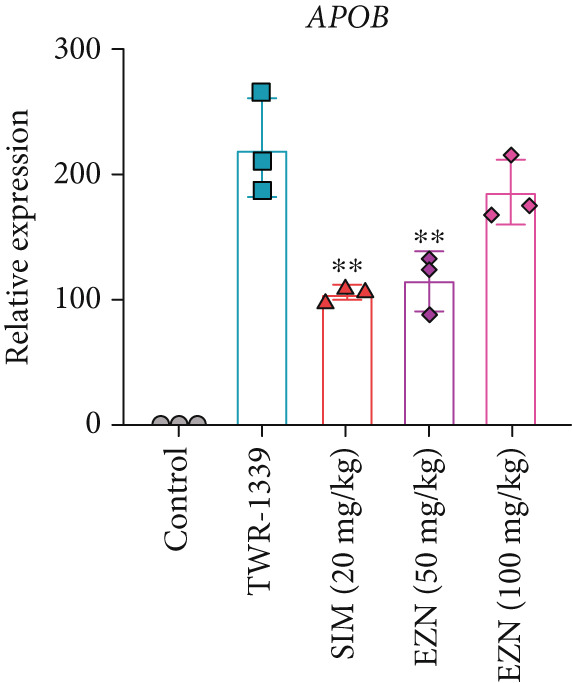
(c)

**Figure:**
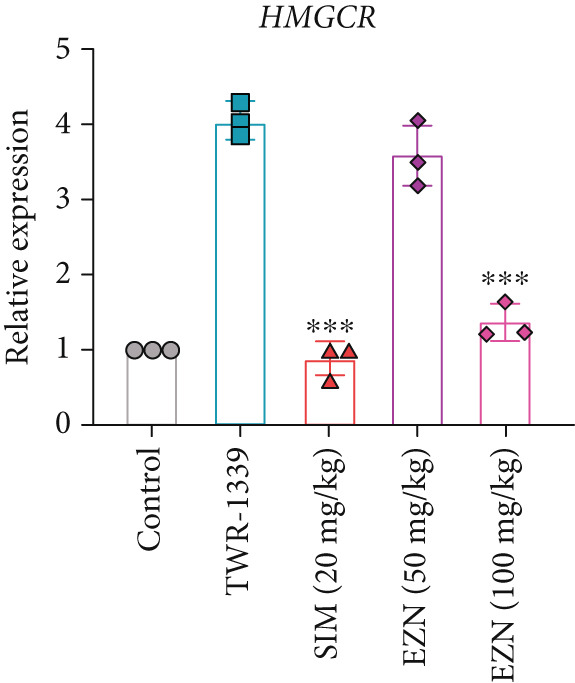
(d)

**Figure:**
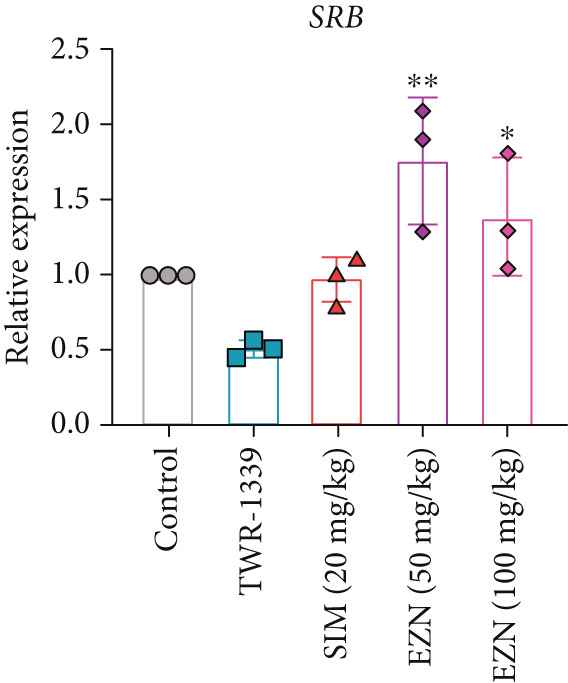
(e)

**Figure:**
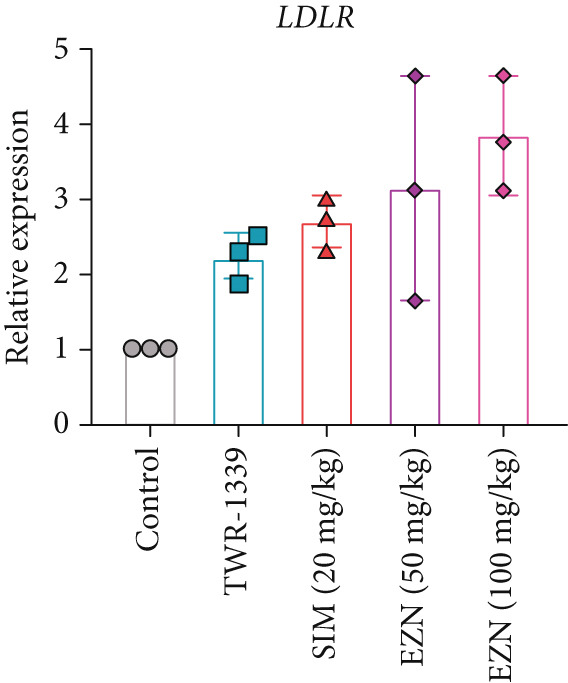
(f)

Taken together, these findings highlight EZN’s broad modulatory role on lipid metabolism genes, particularly its strong capacity to upregulate PPAR‐*α* and SRB, and suppress HMGCR, PCSK9, and APOB.

## 4. Discussion

The present study provides compelling evidence that EZN, a naturally occurring flavan‐3‐ol, exerts significant anti‐hyperlipidemic effects in a TWR‐1339‐induced hyperlipidemic rat model. The findings align with its multi‐target modulation of lipid metabolism pathways, including upregulation of PPAR‐*α*, SRB, and LDLR, alongside the suppression of PCSK9 and HMGCR. These multi‐target effects position EZN as a promising candidate for managing dyslipidemia, particularly in cases where conventional therapies exhibit limitations. Below, we present a detailed examination of these mechanisms, their physiological consequences, and their therapeutic implications within the context of current pharmacological strategies for hyperlipidemia.

The most pronounced effect of EZN was its ability to significantly reduce serum TG and VLDL levels. This observation aligns with EZN’s potent upregulation of PPAR‐*α*, a nuclear receptor that serves as the master regulator of fatty acid *β*‐oxidation [38]. PPAR‐*α* activation induces the transcription of genes encoding lipoprotein lipase (LPL), acyl‐CoA oxidase (ACOX), and carnitine palmitoyltransferase‐1 (CPT‐1) [39]. LPL hydrolyzes circulating TG‐rich lipoproteins (chylomicrons and VLDL), while ACOX and CPT‐1 drive mitochondrial and peroxisomal fatty acid oxidation. This dual mechanism explains the reduction in TG levels observed in our high‐dose EZN group, comparable with fibrates (e.g., fenofibrate), which are synthetic PPAR‐*α* agonists [40]. Notably, EZN’s natural origin may circumvent the hepatotoxicity and myopathy risks associated with fibrates [41], though further safety studies are warranted.

EZN’s hypocholesterolemic effects were equally striking, with a reduction in LDL, which can be attributed to its dual modulation of the PCSK9‐LDLR pathway. PCSK9, a serine protease secreted by hepatocytes, binds to LDLR and promotes its lysosomal degradation, thereby reducing hepatic cholesterol clearance [42]. Our data show that EZN downregulates PCSK9 expression, mirroring the effects of monoclonal antibody inhibitors (e.g., alirocumab). However, unlike these biologics that are costly and require subcutaneous administration, EZN offers an orally active, small‐molecule alternative. Concurrently, EZN upregulated LDLR expression, enhancing LDL particle endocytosis. This contrasts with statins, which increase LDLR expression but paradoxically upregulate PCSK9 as a compensatory response [43]. EZN’s ability to concurrently suppress PCSK9 and induce LDLR suggests a synergistic mechanism that could outperform statins in certain dyslipidemic phenotypes.

Although statins directly inhibit HMGCR (the rate‐limiting enzyme in cholesterol synthesis), EZN exhibited a more nuanced effect. This partial inhibition may explain why EZN lowered TC by 31% compared with the 30%–35% reduction typical of high‐dose statins [44]. However, this moderate effect could be advantageous in avoiding statin‐associated adverse effects like myopathy or diabetes [45]. Importantly, EZN’s anti‐hyperlipidemic actions extend beyond HMGCR inhibition, leveraging PPAR‐*α* and PCSK9 pathways to achieve comparable efficacy through multi‐target synergy.

The upregulation of SRB at 50 mg/kg (*p* < 0.01) underscores EZN’s role in reverse cholesterol transport (RCT). SRB facilitates HDL‐mediated cholesterol efflux from peripheral tissues to the liver [46], explaining the significant HDL elevation. The lack of SRB upregulation at 100 mg/kg suggests dose‐dependent saturation or competing pathways, highlighting the need for optimized dosing strategies.

Several natural compounds have shown multi‐target lipid‐lowering effects in preclinical studies like EZN. For instance, berberine, an alkaloid, lowers LDL via transcriptional upregulation of LDLR and downregulation of PCSK9 [47, 48]. Similarly, the flavonoid naringenin from citrus fruit activates PPAR‐*α* and accordingly lowers the production of VLDL/TG and ameliorates dyslipidemia in various rodent models suggesting the potential complementarity of PPAR‐*α* [49, 50]. Quercitin‐3‐glucoside has shown an improvement in LDL clearance via differential regulation of LDLR/PCSK9 axis [51]. Moreover, curcumin and its related compounds have shown to improve lipid profile in animal models owing to their HMGCR inhibitory effects [52].

Although EZN improved lipid profiles, its hepatotoxic potential evidenced by elevated AST requires scrutiny. Statins are notorious for transiently increasing liver enzymes due to mitochondrial stress and hepatocellular leakage [53]. However, EZN’s hepatotoxic effects were milder than SIM, with ALP levels rising significantly only at 100 mg/kg (*p* < 0.05). The AST elevation, though statistically significant, remained within subclinical ranges, suggesting reversible stress rather than overt damage. The dichotomy between ALT and ALP trends is intriguing. Though SIM increased ALT (a marker of hepatocellular injury), EZN reduced it, particularly at 50 mg/kg. This divergence implies EZN may mitigate hepatocellular damage while inducing cholestatic effects (via ALP elevation). Such hepatoprotective properties could stem from EZN’s antioxidant activity, as previously reported in cisplatin‐induced renal injury models [54].

Moreover, the molecular docking component of this study provided mechanistic validation for EZN predicted interactions with key lipid‐regulating targets. The structural complementarity between EZN and proteins such as HMGCR, PCSK9, PPAR‐*α*, and LDLR reinforces the systems pharmacology and gene network findings and underlines EZN’s potential as a multi‐target agent in the management of dyslipidemia. In silico docking confirmed that EZN can engage directly with lipid‐regulatory proteins involved in cholesterol biosynthesis, lipoprotein uptake, and fatty acid metabolism. These docking interactions are not merely theoretical; they align with the molecular mechanisms previously established for effective hypolipidemic agents. For example, plant‐based polyphenols have been shown to bind HMGCR, partially inhibiting cholesterol synthesis through structurally favorable interactions that are less potent but potentially safer than statins [44, 45].

The structural docking of EZN with PCSK9 is particularly noteworthy. Small molecules capable of binding PCSK9 and inhibiting its interaction with LDLR offer a promising alternative to costly monoclonal antibodies [15, 42, 43]. Docking results suggest that EZN has the potential to exert such an effect, which supports its relevance in both statin‐tolerant and statin‐intolerant populations. Likewise, the observed binding of EZN to PPAR‐*α* provides a plausible structural basis for the modulation of fatty acid oxidation, consistent with known actions of PPAR‐*α* agonists such as fibrates [40]. Importantly, flavonoids like EZN may avoid the hepatic and muscular adverse effects that limit the use of synthetic agents in this class [41]. These docking findings validate the pharmacological targets predicted via network pharmacology and support EZN’s pleiotropic role in lipid regulation. The convergence of network‐based predictions, experimental gene expression changes, and molecular docking interactions provides a coherent mechanistic framework, positioning EZN as a promising natural candidate in hyperlipidemia management.

Overall, EZN’s multi‐target mechanism, simultaneously modulating PPAR‐*α*, PCSK9, and SRB, distinguishes it from single‐pathway drugs like statins or fibrates. This polypharmacology could enhance efficacy, particularly in resistant or mixed hyperlipidemia. For instance, statins primarily lower LDL via HMGCR inhibition but have limited impact on TG or HDL [39], whereas EZN addresses multiple lipid fractions. Furthermore, EZN’s anti‐inflammatory properties, demonstrated in carrageenan‐induced edema models [55], may synergize with lipid‐lowering to attenuate atherosclerosis, a process driven by inflammation and lipid deposition [56].

## 5. Conclusion

EZN emerges as a promising multi‐target agent for hyperlipidemia, leveraging PPAR‐*α* activation, PCSK9 inhibition, and SRB upregulation to improve lipid profiles. Though its hepatotoxic effects are milder than statins, they underscore the need for cautious dosing and further safety profiling. The study’s acute model and rodent‐specific limitations necessitate validation in chronic settings and human trials. If successful, EZN could complement existing therapies, particularly for patient intolerant to statins or those requiring HDL‐boosting interventions. As the global burden of hyperlipidemia grows, natural compounds like EZN offer a compelling avenue for safer, holistic management of cardiovascular risk.

## 6. Future directions

In order to translate these findings, long‐term preclinical studies, using diet‐induced or genetically modified animal models, should be performed to assess EZN’s efficacy, tolerability and chronic safety. Combination therapies with low‐dose statins or fibrates may offer enhanced lipid lowering effects together with reduced high‐dose monotherapy‐related adverse events. Finally, understanding of gut microbiota‐mediated metabolism of EZN may provide new insights including the modulation of bile acid signaling, short‐chain fatty acid production, and intestinal cholesterol handling. Together, these approaches will provide impetus for the continued development of EZN as a natural multi‐target therapeutic agent for hyperlipidemia.

## Conflicts of Interest

The authors declare no conflicts of interest.

## Author Contributions

**Saud O. Alshammari, Nazifa Shahzad**: data curation, formal analysis, methodology, investigation, resources, software, validation, visualization, writing – original draft); **Saud O. Alshammari**: funding acquisition; **Muhammad Nasir Hayat Malik**: conceptualization, data curation, formal analysis, methodology, project administration, resources, supervision, validation, visualization, writing – review & editing; **Qamar A. Alshammari, Abdulkarim Alshammari, Bassam S. M. Al Kazman, Muhammad Atif**: data curation, formal analysis, methodology, investigation, resources, software, validation, visualization, writing – review & editing); **Gideon F. B Solre**: conceptualization, data curation, formal analysis, methodology, project administration, resources, software, writing – review & editing. Saud O. Alshammari, Nazifa Shahzad, and Muhammad Nasir Hayat Malik contributed equally.

## Funding

This study was supported by Deanship of Scientific Research at Northern Border University, NBU‐FFR‐2025‐177‐02.

## Data Availability

All relevant data supporting the findings of this study are included within the main manuscript.
